# Sphaerodoridae (Annelida) of the deep Northwestern Atlantic, including remarkable new species of *Euritmia* and *Sphaerephesia*

**DOI:** 10.3897/zookeys.615.9530

**Published:** 2016-09-07

**Authors:** María Capa, Karen J. Osborn, Torkild Bakken

**Affiliations:** 1 Norwegian University of Science and Technology, NTNU University Museum, NO-7491 Trondheim, Norway; 2 Smithsonian National Museum of Natural History, Dept. of Invertebrate Zoology, 10th and Constitution Ave. NW, Washington, D.C. USA

**Keywords:** Generic synonymy, Amacrodorum, Ephesiopsis, epithelial tubercles, NW Atlantic, shelf, slope

## Abstract

Sphaerodoridae (Annelida) is a seeming uncommon and minimally diverse group of polychaetes in the northwestern Atlantic, with only seven species reported from the United States, and none from the eastern coast of Canada, before the present study. Review of the large Smithsonian collection (National Museum of Natural History, Washington) revealed the presence of two morphologically extraordinary undescribed species and added a new record to the north-western Atlantic region. *Euritmia carolensis* sp. n. is characterised by bearing approximately 20 sessile spherical papillae arranged in three transverse rows per segment, ventrum with 4–6 larger papillae near the parapodial bases and parapodia without papillae; bearing 4–5 simple chaetae that are enlarged subdistally. *Sphaerephesia amphorata* **sp. n.** is distinguished from other congeners in the presence of four longitudinal rows of sessile, bottle-shaped macrotubercles with exceptionally long digitiform terminal papilla, and parapodia with four rounded and small papillae, bearing 4–7 compound chaetae, with blades 7–11 times as long as wide. Other encountered species are also herein re-described, including intraspecific variation and updated iconography. Comparison of material also allowed some systematic changes in the group, including the synonymisation of the genus *Amacrodorum* with *Euritmia*, and the transfer of *Ephesiopsis shivae* to *Ephesiella*. A key to the species reported from the Northwestern Atlantic is provided.

## Introduction

The northwest Atlantic (considered herein as the continental shelf and slope areas off Atlantic Canada and New England) is a relatively well-studied area in terms of its benthic polychaete fauna (e.g. Pettibone 1952, Hartman 1965, Blake 1971, Hartman and Fauchald 1971, Maciolek 1981, 2000, Hilbig and Blake 1991, Frame 1992, Schaff et al. 1992, Blake and Grassle 1994, Hilbig 1994). The dominant taxonomic group in these environments, representing nearly half of the species of the infauna, is the Annelida (Hilbig and Blake 1991, Grassle and Maciolek 1992, Schaff et al. 1992, Blake and Grassle 1994). Sphaerodoridae, a polychaete family typically known to inhabit deep sediments, were neither particularly abundant nor diverse (Hilbig and Blake 1991, Schaff et al. 1992, Blake and Grassle 1994). Seven nominal species have been reported from the area (Table 1), together with probably another eight undescribed species (Hilbig and Blake 1991). Surprisingly, all the sphaerodorids known to the northwest Atlantic have been reported from the United States waters, and none has yet been recorded from Canada (Pocklington and Tremblay 1987, Carr 2012).

**Table 1.** Species recorded in northwestern Atlantic shelf, slope and abyssal depths.

Species	Type locality
*Clavodorum atlanticum* Hartman & Fauchald, 1971	Northwest of Bermuda, 37°59.2'N, 69°26.2'W, 3834 m depth.
*Ephesiella macrocirris* Hartman & Fauchald, 1971	Off New England, 39°46.5'N, 70°43.3'W, 1470–1330 m depth.
*Ephesiella mixta* Hartman & Fauchald, 1971	Off New England, 38°33'N, 68°32'W, 3753 m depth.
*Sphaerororidium minutum* (Webster & Benedict, 1887)	Off New England, mainly shelf depths
*Sphaerodoropsis corrugata* Hartman & Fauchald, 1971	Off New England, 39°56'30"N, 70°39'54"W, 400 m depth.
*Sphaerodoropsis elegans* Hartman & Fauchald, 1971	Off Brazil, 00°03.0'S, 27°48.0'W, 3730–3783 m depth.
*Sphaerodoropsis longipalpa* Hartman & Fauchald, 1971	Bermuda Slope, 32°16'36"N, 64°36'18"W, 1700 m depth.

Revision of the sphaerodorids deposited at the Smithsonian collection, National Museum of Natural History, Washington, revealed undescribed species fitting the definition of *Euritmia* Sardá-Borroy, 1987 and *Sphaerephesia* Fauchald, 1972, but presenting noteworthy attributes never described in any member of either of these two groups. *Euritmia hamulisetosa* Sardá-Borroy, 1987, type species of the genus, was described from shallow subtidal environments in southern Spain. *Euritmia capense* (Day, 1963) is so far considered the only congener and reported from South Africa (Day 1963, Sardá-Borroy 1987). Members of this genus are characterised by the absence of macrotubercles (i.e. large tubercles arranged in longitudinal rows) on the dorsum, absence of microtubercles (i.e. tubercles provided with a terminal papillae and a basal collar) on the dorsum, presence of epithelial papillae (smaller than macrotubercles) and arranged in various transversal rows per segment, and the presence of only simple chaetae on every parapodia (Sardá-Borroy 1987, Fauchald 1974). These attributes are all shared by *Amacrodorum* Kudenov, 1987a (Capa et al. 2014), which gives reasons to investigate the validity of both genera.

Members of *Sphaerephesia*, a genus with nine nominal species described to date, are recognised by the presence of macrotubercles with a terminal papilla and compound chaetae (Fauchald 1972, 1974; Capa and Bakken 2015). However, the terminal papillae are not often evident and instead, pear shaped tubercles have been observed in some species (see Capa et al. 2014, Capa and Bakken 2015). This is not the case for the new species of *Sphaerephesia* described herein, which is provided with an exceptional digitiform papilla on all dorsal macrotubercles.

For this study, specimens of previously described species from the area were also examined, allowing re-description of *Ephesiopsis guayanae* Hartman & Fauchald, 1971, *Sphaerodoridium minutum* (Webster & Benedict, 1887) and *Sphaerodoropsis corrugata* Hartman & Fauchald, 1971. Each of these species is also peculiar and inhabit the northwestern Atlantic. In the process of re-describing these species we comment on the morphological intraspecific variability observed.

This study does not intend to represent an exhaustive taxonomic account of the sphaerodorid fauna of the northwest Atlantic. Instead, the aim is to highlight the need of further benthic surveys and taxonomic revisions of the material housed in museum collections in order to increase the knowledge of the biodiversity inhabiting the northwestern Atlantic Ocean floor, including the description of new species with remarkable morphological characteristics that may assist in understanding the morphological variation of the family.

## Methods

Specimens deposited in the collections of the Smithsonian Institution, National Museum of Natural History, Washington (USNM) mainly collected in the Atlantic Slope and Rise Program (ASLAR) were examined. Holotypes and comparative material from other institutions (Australian Museum
(AM), Sydney; Zoologisches Museum
(ZMH), Hamburg; Natural History Museum of Los Angeles County
(LACM-AHF), Los Angeles, and Museu de Zoologia Universidade de São Paulo
(MZSP), São Paulo) were also studied. The material was fixed in formalin and preserved in 70–80% ethanol. Specimens were examined under dissecting and compound microscopes.

Methylene-blue staining was used to highlight glandular areas and papillae by immersing selected specimens in 70–80% ethanol with some dissolved crystals of the compound for several minutes. Micrographs were taken with a Leica DFC 420 camera attached to a Leica MZ 16A stereo microscope and a Leica DM 6000B compound microscopes (Leica Microsystems, Wetzlar, Germany). Stacks of multi-focus shots were merged into a single photograph to improve resolution with Leica Application Suite v3.7 software (Leica Microsystems, Wetzlar, Germany). Some parapodia were mounted on microscope slides with glycerine.

Scanning electron micrographs were taken on specimens after dehydrating them in a series of 70, 80, and 90% ethanol and series of mixtures of absolute ethanol and Hexamethyldisilazane (HMDS) with the following ratios 2:1, 1:1, 1:2, and then into pure HMDS. The prepared samples were mounted on holders, sputter-coated with gold (10 nm thickness). The micromorphology and topography were determined using a Philips FEI INSPECT (Hillsboro, Oregon, USA) scanning electron microscope (SEM) at the Museo Nacional Ciencias Naturales (Madrid, Spain) and a JEOL-JSM-6480 SEM at the Cellular and Molecular Imaging Core Facility (CMIC) of the Faculty of Medicine of the Norwegian University of Science and Technology
(NTNU). The samples were observed with the Back Scattering Electron Detector (BSED) with a resolution at high vacuum of 4.0 nm at 30 kV. The accelerating voltage was 30 kV and working distance of 10 mm to the detector.

A key for species identification was generated after consideration of the species reported from the northwestern Atlantic (with an asterisk) and other from adjacent geographic regions such as the Gulf of Mexico and the Caribbean (considering recent reviews e.g. Kudenov 1987b, 1994, Salazar-Vallejo 1996, Fauchald et al. 2009) in view that some of these species, especially those typical from deeper environments, could be present in the area. The features used in this key correspond mostly to original descriptions except for the material reviewed in the present study. Species originally described in distant disjunct geographic areas and whose identifications are dubious have been left out of the key (e.g. *Sphaerodoridium claparedii* (Greeff, 1866) and *Sphaerodorum ophiurophoretos* Martin & Alvà, 1988 or those currently not considered belonging to the family (e.g members of *Levidorum* Perkins, 1987 or *Sphaerodoridium guilbaulti* Rullier, 1974).

**Figure 1.**
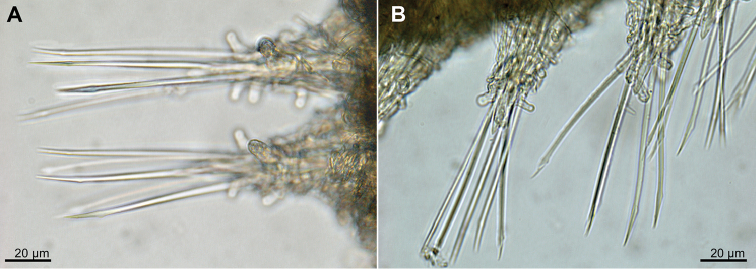
*Ephesiopsis guayanae* paratypes LACM-AHF POLY TYPE 943, micrographs. **A** Mid-body chaetigers and chaetae (apparently simple), ventral view **B** Mid-body chaetigers with compound and simple chaetae, dorsal view.

Abbreviations used on the figures: 1st, first chaetiger; al, acicular lobe; ap, antenniform papillae; eg, egg; la, lateral antenna; ma, median antenna; mc, macrotubercle; mi, microtubercle; mo, mouth; no, nuchal organ; pa, palp; tc, tentacular cirrus; vc, ventral cirrus.

## Systematics

### Ephesiopsis

Taxon classificationAnimaliaPhyllodocidaSphaerodoridae

Hartman & Fauchald, 1971

Ephesiopsis Hartman & Fauchald, 1971: 68; Fauchald 1974: 270; Rizzo 2009: 62–63 (in part); Capa et al. 2014.

#### Type species.

*Ephesiopsis guayanae* Hartman & Fauchald, 1971.

#### Diagnosis.

Body long and slender. Two longitudinal rows of macrotubercles, one pair per segment, absent on first chaetiger. Macrotubercles sessile, with terminal papillae. Two longitudinal rows of microtubercles, one pair per segment, running parallel between macrotubercles. Additionally, papillae arranged in 4–5 transverse rows on dorsum and ventrum. Prostomial and peristomial appendages short, spherical or digitiform. Parapodia from chaetiger 2 with both simple and compound chaetae; hooks on first chaetiger absent or present.

#### Remarks.

The genus was originally erected and justified by the presence of both simple and compound chaetae in every chaetiger (Hartman and Fauchald 1971, Fauchald 1974), a condition different to that found in members of *Ephesiella* Chamberlin, 1919, with only compound or pseudo-compound chaetae (Moore 1909, Fauchald 1974, Capa and Bakken 2015, Capa et al. 2014, 2016), or *Sphaerodorum* Örsted, 1843, with typically only simple chaetae (Fauchald 1974, Capa et al. 2014, 2016). Otherwise, these three genera are very similar and they all share the general body shape, with slender bodies, the number and arrangement of epithelial tubercles, with two longitudinal rows of macrotubercles with a terminal papilla, two longitudinal rows of microtubercles, and about 3‒4 transverse rows of smaller papillae on each segment. The re-examination of the types of the type species, *Ephesiopsis guayanae* Hartman & Fauchald, 1971, confirms the presence of sub-distally widened simple chaetae, with tapering tips and straight edges, and with apparently no sign of being compound under the light and compound microscopes (Fig. 1A, B); while an additional chaeta in same parapodia is compound, with thinner blades and with more rounded edges. It is unclear at this point, if the presence of both compound and apparently simple chaetae justifies the validity of the genus and further analyses should be performed to elucidate this issue.

An examination of the types of the recently described *Ephesiopsis shivae* Rizzo, 2009 from Brazil revealed that the specimens do not show the typical generic attributes (Rizzo 2009, Fig. 3A–C, E–F). The chaetae considered in the original description as simple seem to be compound chaetae that have lost the blades, as it has been observed in many specimens of *Ephesiella* spp. Moreover, *Ephesiella shivae* was described as having a pygidium with four macrotubercles (Rizzo 2009), but the examined Brazilian specimens showed the typical two dorsal macrotubercles and the ventral digitiform pygidial cirrus. Based on this observation, we propose that *Ephesiella shivae* is transferred to *Ephesiella* and conclude that *Ephesiopsis* is monotypic.

### Ephesiopsis guayanae

Taxon classificationAnimaliaPhyllodocidaSphaerodoridae

Hartman & Fauchald, 1971

Figs 1 , 2

Sphaerodorum sp. C.– Hartman 1965: 96, pl. 14, Figs A–B.
Ephesiopsis guayanae Hartman & Fauchald, 1971: 68–69, Pl 33, figs A–G.

#### Material examined.

**Holotype**: LACM-AHF POLY TYPE 942, off Dutch Guayana Surinam, 07°52'N, 54°31.5'W, 520–550 m, coll. Woods Hole Oceanographic Institution, 25 Apr 1963. **Paratype**: same collection information LACM-AHF POLY TYPE 943 (1 ind.).

#### Additional material.

USNM 1001772 (2 ind.), Georges Bank, 40°57.21'N, 066°13.68'W, coll. MMS Collections, Atlantic Slope and Rise Program, ASLAR, 25 Jul 1986; USNM 1001773 (1 ind.) off New Jersey, 38°35.98'N, 072°52.86'W, 2195 m, coll. MMS Collections;

USNM 1001777 (1 ind.), off New Jersey, United States, 2150 m, 1 Dec 1984, coll. MMS Collections, Atlantic Slope and Rise Program, ASLAR; USNM 1001713 (1 ind.), off Cape Lookout, North Carolina, 34°11.16'N, 75°38.98'W, 2006 m, coll. MMS Collections, Atlantic Slope and Rise Program, ASLAR, 14 Jul 1984; USNM 1001780 (1 ind.), off Delmarva, 37°51.58'N, 73°19.914'W, 2100 m, coll. MMS Collections, Atlantic Slope and Rise Program, ASLAR, 30 Nov 1984; USNM 1001781 (1 ind.), off New Jersey, 38°29.28'N, 72°42.11'W, 2507 m, coll. MMS Collections, Atlantic Slope and Rise Program, ASLAR, 4 Dec 1984; USNM 1001782 (1 ind.), off New Jersey, 38°29.23'N, 72°42.19'W, 2505 m, coll. MMS Collections, Atlantic Slope and Rise Program, ASLAR, 18 May 1985; USNM 1001783 (1 ind.), off New Jersey, 38°29.23'N, 72°42.19'W, 2505 m, coll. MMS Collections, Atlantic Slope and Rise Program, ASLAR, 18 May 1985; USNM 1001789 (1 ind.), Baltimore Canyon, Maryland, 37°53.76'N, 73°44.76'W, 1499 m, coll. MMS Collections, Atlantic Slope and Rise Program, ASLAR, 15 Nov 1985.

#### Comparative material.

*Ephesiopsis shivae*, holotype MZSP883 24°07.637'S 45°51.895'W, 09 Jan 1998, Sta. 6661, 147 m, Santos/São Paulo to Ilha Grande Bay/Rio de Janeiro; paratypes MZSP1031 (2 ind.), 24°07.637'S, 45°51.895'W, 09 Jan 1998, Sta. 6661, 147 m, Santos/São Paulo to Ilha Grande Bay/Rio de Janeiro.

#### Diagnosis.

Palps and lateral antennae digitiform, median antenna shorter. Tentacular cirri ellipsoid. Parapodia with 4–6 parapodial papillae; compound chaetae with blades 1.5–2.5 times as long as maximum width on mid-body chaetigers, simple chaetae wider and with angular silhouette; hooks present on first chaetiger.

#### Re-description.

*Measurements and general morphology*. Holotype 2.2 mm long, 0.2 mm wide, with 26 chaetigers, divided in two. Body elongated, sub-quadrangular in section, with slightly convex dorsum. Anterior end bluntly rounded, slightly narrowing along posterior segments. Segmentation inconspicuous, tegument with transverse wrinkles. Preserved specimen lacking pigmentation.

**Figure 2.**
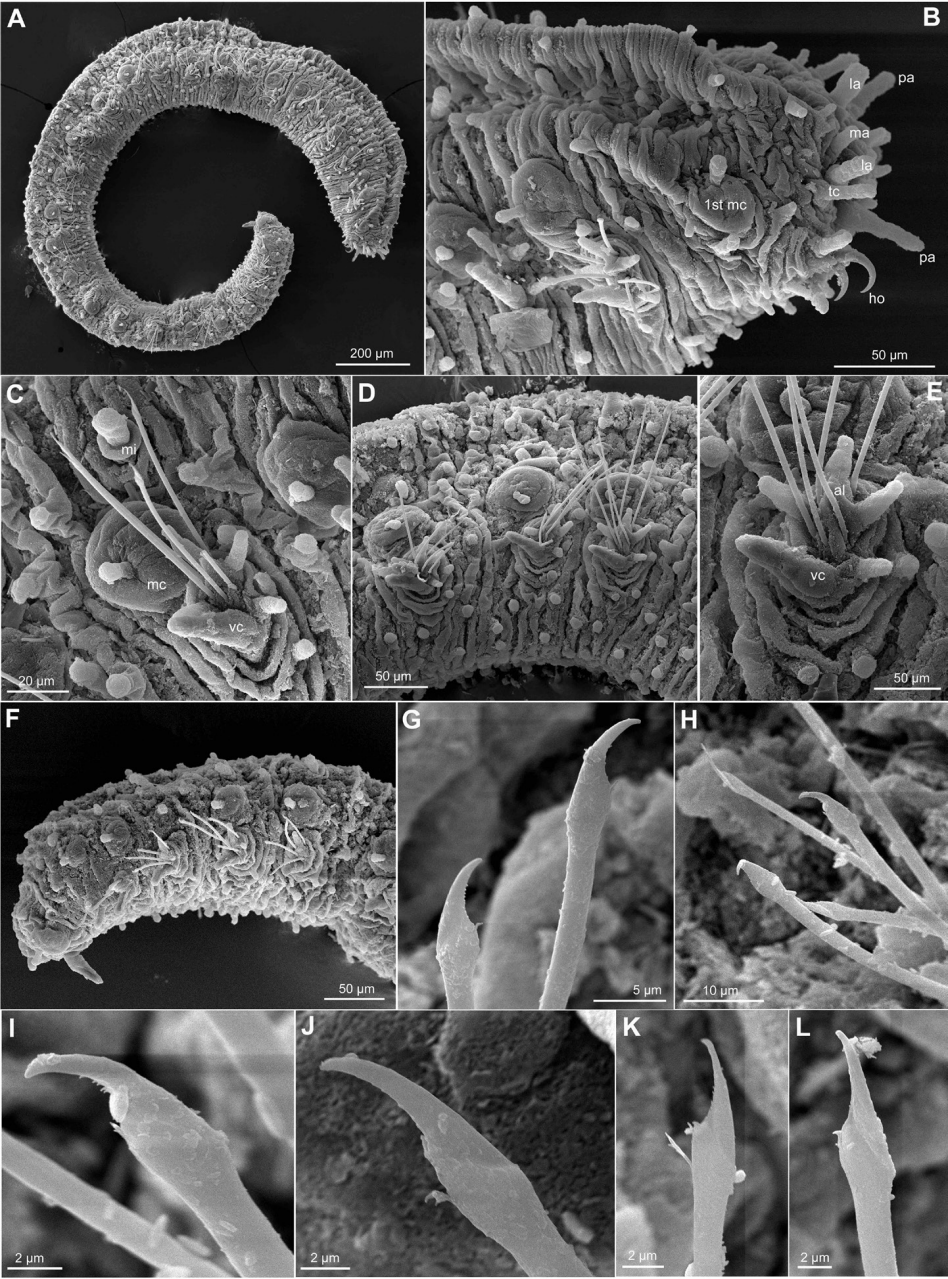
*Ephesiopsis guayanae*, USNM 1001777, SEM. **A** Whole specimen, side view **B** Anterior end, side view **C** Chaetiger 3, with parapodia, macrotubercle and microtubercle, side view **D** Mid-body segments, side view **E** Mid-body parapodium showing detail of parapodial papillae, acicular lobe and ventral cirrus **F** Posterior chaetigers and pygidium with paired dorsal cirri and ventral digitiform anal cirrus, side view **G** Chaetae chaetiger 4 **H** Chaetae mid-body fascicle **I–L** Chaetae mid-body chaetigers. Abbreviations: 1^st^ mc, macrotubercle from first chaetiger; al, acicular lobe; la, lateral antenna; ma, median antenna; mc, macrotubercle; mi, microtubercle; mo, mouth; no, nuchal organ; pa, palp; tc, tentacular cirrus; vc, ventral cirrus.

*Head*. Prostomium with five short appendages, including a pair of digitiform palps in ventral-most position, a pair of lateral antennae, similar in shape and size to palps, and a median antenna, shorter (one third) and thinner than lateral antennae (Fig. 2A, B). A pair of tentacular cirri shorter than lateral antennae and palps. A few rounded (about six) small papillae confined by head appendages.

*Tubercles*. First chaetiger with two dorsal macrotubercles; microtubercles absent (Fig. 2B). Following chaetigers each with two dorsal macrotubercles arranged in two dorso-lateral longitudinal rows, and two microtubercles forming two longitudinal rows between the macrotubercles (Fig. 2B–D, F). Macrotubercles sessile and spherical each provided with a digitiform terminal papilla (Fig. 2B–D, F); with groups of pores around terminal papilla. All macrotubercles similar in shape and size. Microtubercles with digitiform terminal papilla generally longer than collar (Fig. 2C). Spherical or ellipsoid papillae over dorsum, arranged in 3–4 transversal rows per chaetiger, with around 15–20 papillae on each mid-body chaetigers. Ventral surface with spherical papillae, arranged in four more or less regular transversal rows, with about 20 per segment, in mid-body; numbers decreasing towards posterior end (Fig. 2F). Body epithelium with ellipsoid granules.

*Parapodia*. Parapodia sub-conical, increasing in size towards chaetiger 3 (Fig. 2B), around 1–3 times longer than wide (Fig. 2C–E). Acicular lobe projecting distally anterior to chaetae, resembling other parapodial papillae or slightly longer (Fig. 2E). Ventral cirri digitiform projecting as long as acicular lobe on anterior segments or shorter in mid-body and posterior ones (Fig. 2C–E). Mid-body parapodia with 4–5 ellipsoid to digitiform papillae, all similar in size, in addition to the acicular lobe: 2–3 anterior, 1–2 posterior-ventral (Fig. 2C–E).

*Chaetae*. First chaetiger with two pairs of hooks, one pair on each parapodia together with elongate simple chaetae. One compound and 2–4 simple chaetae in all chaetigers, arranged in a curved transverse row around acicular lobe (Figs 1A, B, 2G–L). First and second chaetigers with slightly serrated long blades, 4–5 times longer than wide. Chaetae from chaetiger 3 with shafts widened sub-distally, a larger distal tooth and fine spinulation; blades twice as long as wide (Fig. 2G–L).

*Pygidium*. Pygidium terminal, with one mid-ventral digitiform anal cirrus and a pair of dorsal anal cirri, similar in shape but slightly smaller than macrotubercles (Fig. 2F).

*Internal features*. A pair of eyes anterior to first chaetiger.

*Reproductive features*. Copulatory organs or eggs not seen in holotype. Paratype with eggs in coelomic cavity.

#### Variation.

The paratype, an incomplete gravid female, is larger than holotype, 3 mm long and 0.25 wide, with 44 chaetigers. The specimen from New Jersey is 2 mm long and 0.25 wide. The number and morphology of chaetae showed variation among the material examined. The holotype possessed two pair of hooks on first chaetiger, absent or not seen in the paratype while the specimen from New Jersey had one pair (Fig. 2B). Additionally, an extra set of thin and simple chaetae, resembling those present in rest of chaetigers were observed only on the holotype. Following chaetigers had fascicles with 3–5 chaetae, one of them clearly compound in the holotype, two in the paratype and up to three in the specimen from New Jersey. Chaetae appearing compound under the light microscope (Fig. 1B) did not clearly seem so under the electron microscope (Fig. 2G, I, J), but at least a faint edge between the shaft and the blade could be noticed. The simple chaetae, probably a result of the fusion of shaft and the blade, show in some cases a different outline, with an angular edge, and a less curved tip (Fig. 2H, L).

#### Remarks.

The most remarkable attribute of this species is the presence of simple chaetae, in all parapodia, that are flat and sub-distally widened and have an angular contour. This differs from other members of the long bodied sphaerodorids (*Ephesiella* and *Sphaerodorum*), where chaetae, simple or compound respectively, present more rounded edges. In this respect, the holotype and paratypes of *Ephesiella guayanae* show some morphological differences, which, if considered as part of the intraspecific variation, may open a discussion of the legitimacy for the genus. These special, simple, sub-distally widened and flat chaetae were not conspicuous in the paratype. The apparently simple chaetae present in both holo- and paratype do not differ much from those pseudocompound chaetae present in other *Ephesiella* (e.g. *Ephesiella brevicapitis* Moore, 1909). With so little material in hand, only two types, it is difficult to conclude on the status of the genus and the identity of the paratype. The species seems not to have been found again, until now.

The chaetae of the specimens collected from sediments of deep New Jersey waters resemble those observed in the paratype of *Ephesiella guayanae*. In every parapodium a group of simple chaetae can be observed, they are not so wide and angular as those present in the holotype, but still they appear to be simple chaetae. Examination of these specimens under SEM revealed a faint oblique mark in the position where an articulation between shaft and blade is expected, and some chaetae seem to be bent at this point. Together these observations suggest that they may be pseudocompound chaetae (Fig. 2G–L). This cannot be addressed in the type material, which needs to be left intact.

#### Type locality.

Surinam, Dutch Guayana, 520–550 m.

#### Distribution.

From Dutch Guayana to New Jersey, 520–2507 m.

### Euritmia

Taxon classificationAnimaliaPhyllodocidaSphaerodoridae

Sardá-Borroy, 1987

Euritmia Sardá-Borroy, 1987: 48; Capa et al. 2014.
Amacrodorum Kudenov, 1987a: 917–918.

#### Type species.

*Euritmia hamulisetosa* Sardá-Borroy, 1987.

#### Diagnosis.

Body short and ellipsoid. Macro- and microtubercles absent; papillae all over body surface and parapodia. Prostomial and peristomial appendages short, spherical or digitiform. Parapodia with simple chaetae, enlarged sub-distally, with serrated cutting edges; hooks absent.

#### Remarks.

This genus was erected to accommodate a small and atypical species with the body covered by numerous papillae and bearing simple chaetae, *Euritmia hamulisetosa* Sardá-Borroy, 1987, from southern Spain, and a species previously described as *Sphaerodorum*, *Euritmia capense* (Day, 1963), from South Africa (Sardá-Borroy 1987). *Euritmia capense* is distinguished from *Euritmia hamulisetosa* in the number, arrangement and type of dorsal epithelial papillae, having two transverse rows of each large and small papillae per segment instead of four rows of similar papillae; and the chaetal morphology with a typical distal spine in *Euritmia hamulisetosa*, absent in *Euritmia capense* (Day 1963, 1967; Sardá-Borroy 1987). A few months later, another genus was erected to accommodate a similar species, *Amacrodorum bipapillatum* Kudenov, 1987a. In addition to the absence of large tubercles and the presence of simple chaetae, this species was characterised by the presence of two kinds of epithelial papillae, spherical and ellipsoid. The latter author was probably not aware of the description of *Euritmia* and also did not notice the diagnostic features of the South African species, similar to *Amacrodorum bipapillatum*. *Amacrodorum bipapillatum* is distinguished from the two previously described species in the number of transverse rows of dorsal papillae and the arrangement of the ellipsoid and hemispherical papillae. The new species described herein shares with the other three species the absence of macro- and microtubercles, the presence of several rows of papillae over the body surface and the presence of distally hooked simple chaetae. For these reasons, all these species are considered as members of the genus *Euritmia* and united under this name. Being an older name than *Amacrodorum* and applying the Principle of Priority (Article 23, International Code of Zoological Nomenclature), *Euritmia* takes priority.

The genus currently gathers four species, distinguished mainly by the shape and arrangement of dorsal and ventral papillae, the number and arrangement of parapodial papillae and the shape of the chaetae (Table 2). This latter attribute has only been studied under SEM in *Euritmia hamulisetosa*, and therefore is the best known in the group; it is probable that the reported smooth chaetae in the other species would show some thin spinulation if observed under high magnification.

**Table 2.** Comparative features of members of *Euritmia*, from original descriptions.

	*Euritmia hamulisetosa*	*Euritmia capense*	*Euritmia bipapillatum*	*Euritmia carolensis* sp. n.
Source	Sardá-Borroy (1987)	Day (1963)	Kudenov (1987a)	this study
Type locality	Cadiz, Spain	Cape Town, South Africa	Alaska, US	South Carolina, US
Depth	Intertidal	?	59 m	799 m
Size (length × width)	0.6×0.125 mm	2.5×0.8 mm	2.1×0.5 mm	0.5×0.2 mm
Number of chaetigers	14	16	16	12
Prostomial appendages	3 pairs + median antenna	not distinguishable	two pairs (longer) + median antenna. Lat ant. with spurs	two pairs (longer) + median antenna
Eyes	two pairs	one pair	one pair	not observed
Shape of dorsal papillae in mid-body	spherical, all similar in size	spherical, two sizes	two kinds and sizes, hemispherical and ellipsoid	two kinds, hemispherical and ellipsoid
Number of dorsal papillae in mid-body	four transversal rows	two transverse rows	three transversal rows	three transversal rows
Ventral papillae	four transversal rows	?	three transversal rows, elliptical and spherical	two transversal rows
Parapodial papillae	one dorsal, one ventral	one dorsal, one ventral and three smaller ones on anterior and posterior surfaces	one on anterior surface	none
Chaetae mophology	serrated, hooked and with distal spine	smooth, hooked	smooth, hooked	smooth or finally serrated, hooked
Number of chaetae per parapodium	six	ten	four or five	five

Moreover, a group of species to date considered belonging to *Sphaerodoropsis* (Group 4, according to Borowski 1994) also show these features, although the chaetae, instead of being simple, could be considered as pseudocompound. These are *Sphaerodoropsis multipapillata* (Hartmann-Schröder, 1974), *Sphaerodoropsis heteropapillata* Hartmann-Schröder, 1987, and *Sphaerodoropsis plurituberculata* Capa & Rouse, 2015. *Commensodorum commensalis* (Lützen, 1961) shares the type of chaetae with *Euritmia*, being typically simple. Nevertheless, *Commensodorum commensalis* has few and small macrotubercles, consisting of four macrotubercles arranged in a simple transverse row per segment. It is still unclear if these taxa are closely related and if the potential fusion of shafts and blades to form simple chaetae (like in *Sphaerodorum*, *Commensodorum*, and *Euritmia*) occurred more than once within the group.

### Euritmia carolensis sp. n.

Taxon classificationAnimaliaPhyllodocidaSphaerodoridae

http://zoobank.org/BCB8304D-75F9-4566-A619-6BA3606B6469

Figs 3 , 4

#### Material examined.

**Holotype**: USNM 1001792, Off Charleston Bump South Carolina United States 32.3944N, 77.0181W, 799 m, coll. Battelle/Woods Hole Oceanographic Institute for BLM/ MMS, Atlantic Slope and Rise Program, ASLAR, 18 Nov 1985. **Paratypes**: USNM 1001791 (1 ind.), off Cape Fear, North Carolina, 33.0806N, 76.4194W, 896 m, 23 Sep 1985; USNM 1001793 (1 ind.), off New Jersey, 38.5944N, 72.8953W, 2024 m, 13 Nov 1985.

#### Type locality.

Off Charleston Bump, South Carolina, 799 m.

#### Diagnosis.

Body short and ellipsoid. Dorsum with approximately 20 sessile spherical papillae arranged in three transverse rows per segment. Ventrum with 4–6 larger papillae near the parapodial bases. Prostomial and peristomial appendages short and ellipsoidal. Parapodia without papillae; with 4–5 simple chaetae with serrated cutting edges, enlarged sub-distally.

#### Description.

*Measurements and general morphology*. Holotype 0.5 mm long, 0.2 mm wide, with 12 chaetigers; gravid female. Body ellipsoid, with strongly convex dorsum and flattened ventrum. Epithelium with transversal wrinkles, segmentation not noticeable (Fig. 4A, B). Pigmentation absent on preserved material.

**Figure 3.**
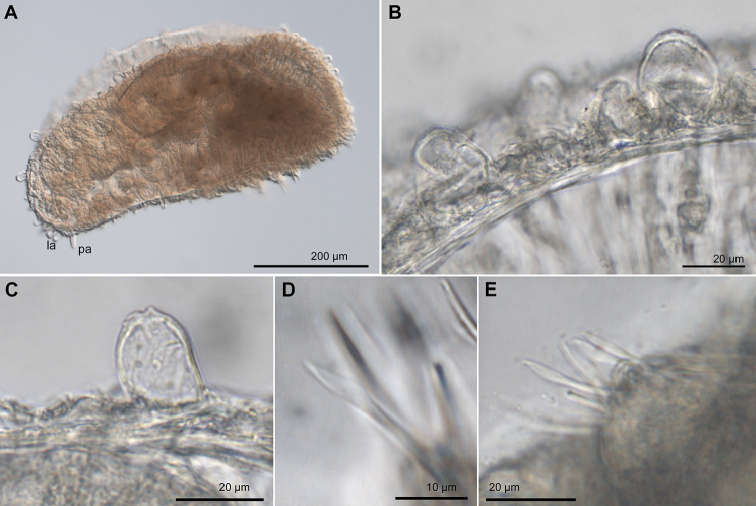
*Euritmia carolensis* sp. n., paratype, USNM 1001791, micrographs. **A** Whole body, side view **B** Dorsal papillae of different sizes, anterior segments **C** Detail of a large dorsal papillae **D** Mid-body simple chaetae **E** Same. Abbreviations: la, lateral antenna; pa, palp.

**Figure 4.**
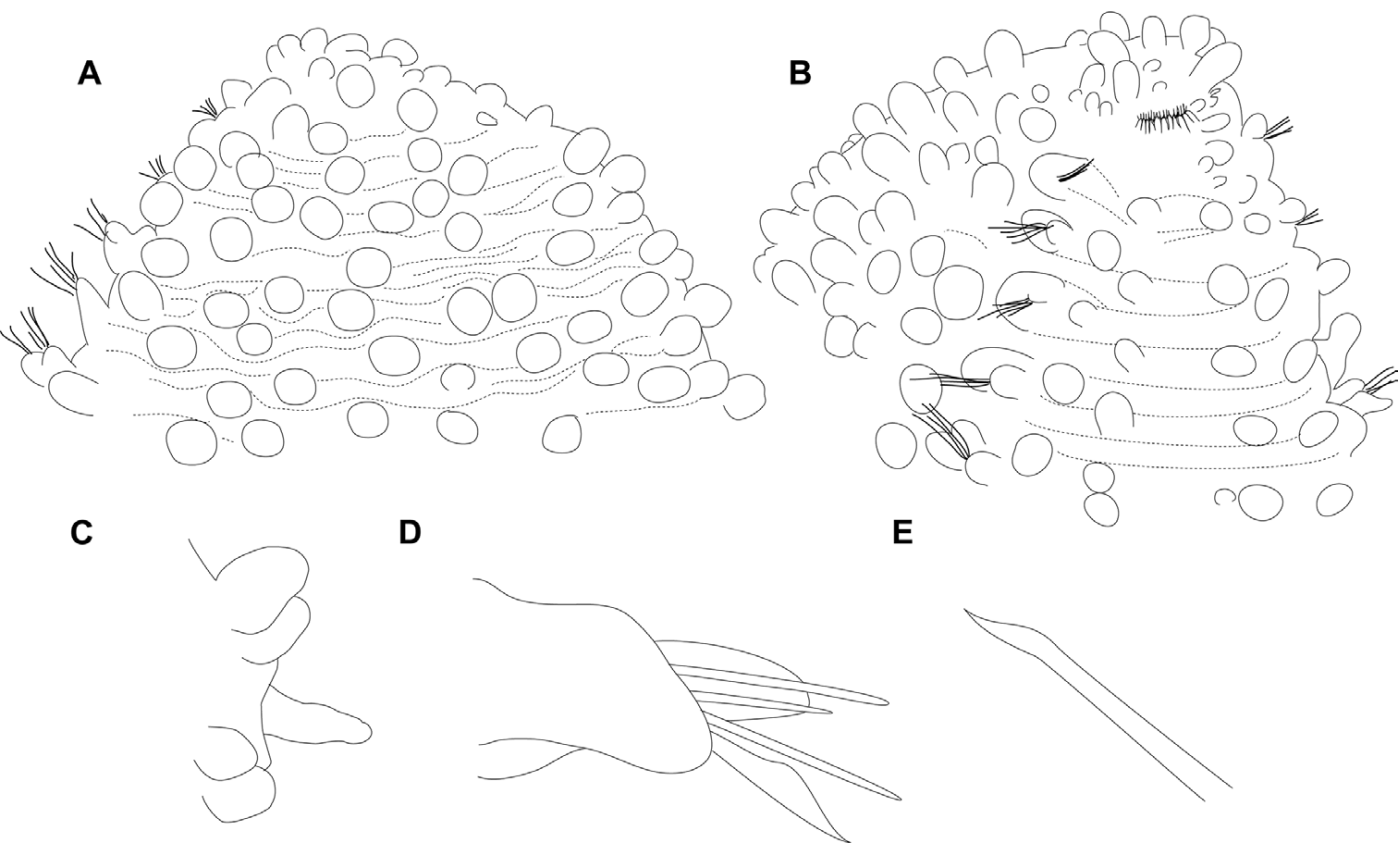
*Euritmia carolensis* sp. n., holotype, USNM 1001792, drawings. **A** Anterior end, dorsal view **B** Same, lateroventral view **C** Pygidium, side view **D** Mid-body parapodium, dorsal view **E** Detail of chaeta.

*Head*. Head fused to first chaetiger (Figs 3A, 4A, B). Prostomial appendages ellipsoid, slightly longer than wide (Figs 3A, 4A). Pair of palps and pair of lateral antennae similar in size, median antenna smaller (Fig. 4B). Dorsal antenniform papillae absent or not conspicuous. Few small hemispherical papillae scattered on head surface, approximately four arranged among palps and antennae. Tentacular cirri, ellipsoid, smaller than palps, similar in size and shape to median antenna (Fig. 4A). Eyes not observed.

*Tubercles*. Dorsum with three transverse rows of papillae per segment (Figs 3A, 4A, B). Papillae sessile, spherical but some larger and ellipsoid papillae over dorsum (Figs 3C–E, 4A), over 20 per segment on mid-body chaetigers. Ventrum with fewer and larger spherical papillae, arranged near parapodial bases in two transverse rows, about 4–6 papillae per segment (Fig. 4B).

*Parapodia*. Parapodia conical, as long as wide in all chaetigers (Fig. 4D), small in anterior chaetigers. Acicular lobe hemispherical; ventral cirri ellipsoid longer than acicular lobe (Fig. 4D). No additional papillae or parapodial appendages.

*Chaetae*. Large, recurved hooks in first chaetiger absent. All parapodia with 4–5 simple chaetae; blades seemingly smooth on cutting edge and slight curved distal tip (Figs 3D, E, 4D, E). One straight acicula per parapodium.

*Pygidium*. Pygidium with two dorsolateral pear-shaped tubercles and single midventral digitiform cirrus, longer than lateral cirri (Fig. 4C).

*Internal features*. Muscular pharynx visible though body wall from chaetiger 2–5.

*Reproductive features*. Copulatory organs not observed. Ovoid eggs measuring 100 µm diameter occupy most of the coelomic cavity.

#### Variation.

The two paratypes lack eggs, other features are very similar.

#### Remarks.

*Euritmia carolensis* sp. n. is distinguished from other congeners by the presence of dorsal papillae arranged in three transverse rows per segment. Most of these are spherical and similar in size but some in a dorsal-most position are elliptical and larger, without a clear distribution pattern. *Euritmia hamulisetosa* is provided with spherical papillae, all similar in size, in four transverse rows per segment; *Euritmia capense* bears two different sizes of spherical papillae each on a single transversal row per segment; and *Euritmia bipapillatum* bears both hemispherical and elliptical papillae in three transversal rows per segment and with a particular zig-zag arrangement (Day 1963, 1967, Sardá-Borroy 1987, Kudenov 1987a) (Table 2). The number and arrangement of ventral papillae are also distinct in the new species, with two transverse rows of 4–6 papillae, mainly arranged near the parapodial bases, while the other species (unknown for *Euritmia capense*) bear numerous papillae with similar arrangement to the dorsum. The parapodia are in all cases conical and small, but only *Euritmia carolensis* sp. n. lacks parapodial papillae; the only appendices present on parapodia being acicular lobe and the ventral cirrus.

#### Etymology.

The epithet of this species refers to the type locality North Carolina.

#### Distribution.

North Carolina to New Jersey (US), from 799 to 2014 m.

### Sphaerephesia

Taxon classificationAnimaliaPhyllodocidaSphaerodoridae

Fauchald, 1972

Sphaerephesia Fauchald, 1972: 97; Magalhães et al. 2011: 40; Capa et al. 2014.

#### Type species.

*Sphaerephesia longisetis* Fauchald, 1972.

#### Diagnosis.

Body short and ellipsoid, some species slender. Four or more longitudinal rows of sessile macrotubercles with terminal papillae. Microtubercles absent (?). Papillae over body surface and parapodia. Prostomial and peristomial appendages short, spherical or digitiform. Parapodia with compound chaetae; hooks absent.

#### Remarks.

There are two species in the genus described as presenting microtubercles (tubercles consisting of a basal collar and a terminal papillae) on the lateral or dorsolateral side of the body, but these differ from those typically present in the long-bodied sphaerodorids (i.e. *Ephesiella*, *Ephesiopsis* and *Sphaerodoridium*).

### Sphaerephesia amphorata sp. n.

Taxon classificationAnimaliaPhyllodocidaSphaerodoridae

http://zoobank.org/9FC1C233-CBF8-4669-BD62-BF4B59CAD091

Figs 5 , 6

#### Material examined.

**Holotype**: USNM 1001815, East of Cape Lookout, North Carolina, United States, north Atlantic, 34°16.32'N, 75°45.498'W, 640 m, 11 Nov 1983, MMS Collections, Atlantic Slope and Rise Program, ASLAR (CRSAP 1 st.1 rep. 2 core 6 sec 0.2). **Paratypes**: USNM USNM 1407168, same sample, different cores (7 ind.). **Additional material.** USNM 1001801 (4 ind.), East of Cape Lookout, North Carolina, United States, North Atlantic, 34°16.002'N, 75°45.967'W, 580 m, 11 Nov 1983; USNM 1001802 (5 ind.), East of Cape Lookout, North Carolina, 34°15.936'N, 75°46.164'W, 583 m, 26 Mar 1984; USNM 1002041 (9 ind., 3 for SEM), East of Cape Lookout, North Carolina, 34°15.816'N, 75°45.786'W, 593 m, 27 Mar 1984; USNM 1002196 (1 ind.), East of Cape Lookout, North Carolina, 34°49.8'N, 75°13.5'W, 2003 m, 20 Jul 1985.

#### Comparative material.

*Sphaerephesia fauchaldi* Kudenov, 1987b, holotype NMNH 102785; *Sphaerephesia longisetis* Fauchald, 1972, holotype AHF POLY 0964; *Sphaerephesia regularis* Böggemann, 2009, holotype ZMH P25498, paratypes ZMH P25497 (4 ind.), ZMH P25499 (4 ind.); *Sphaerephesia similisetis* Fauchald, 1972, paratype AHF POLY 0967 (2 ind.). *Sphaerephesia hutchingsae* Capa and Bakken, 2015, holotype: AM W.42748, East of Malabar, Sydney, New South Wales, Australia, 33°58.717S’, 151°18.00'E, 82 m, 22 Aug 1995; paratypes: east of Malabar, Sydney, New South Wales, 80–28 m. AM W.42717 (1 ind.), AM W.42721 (1 ind.), AM W.42731 (2 ind.), AM W.42749 (1 ind.), AM W.42751 (1 ind.), AM W.42752 (2 ind.), AM W.42758 (1 ind.).

#### Diagnosis.

Four longitudinal rows of sessile, bottle-shaped macrotubercles with long digitiform terminal papilla and 3–4 transverse rows of small spherical papillae per segment. Microtubercles absent. Distance between dorsal-most macrotubercles similar to distance between those and lateral ones. Parapodia with ventral cirri nor surpassing length of acicular lobe and four rounded and small papillae. Parapodia with 4–7 compound chaetae, with thin shafts and blades 7–11 times as long as wide.

#### Description.

**Figure 5.**
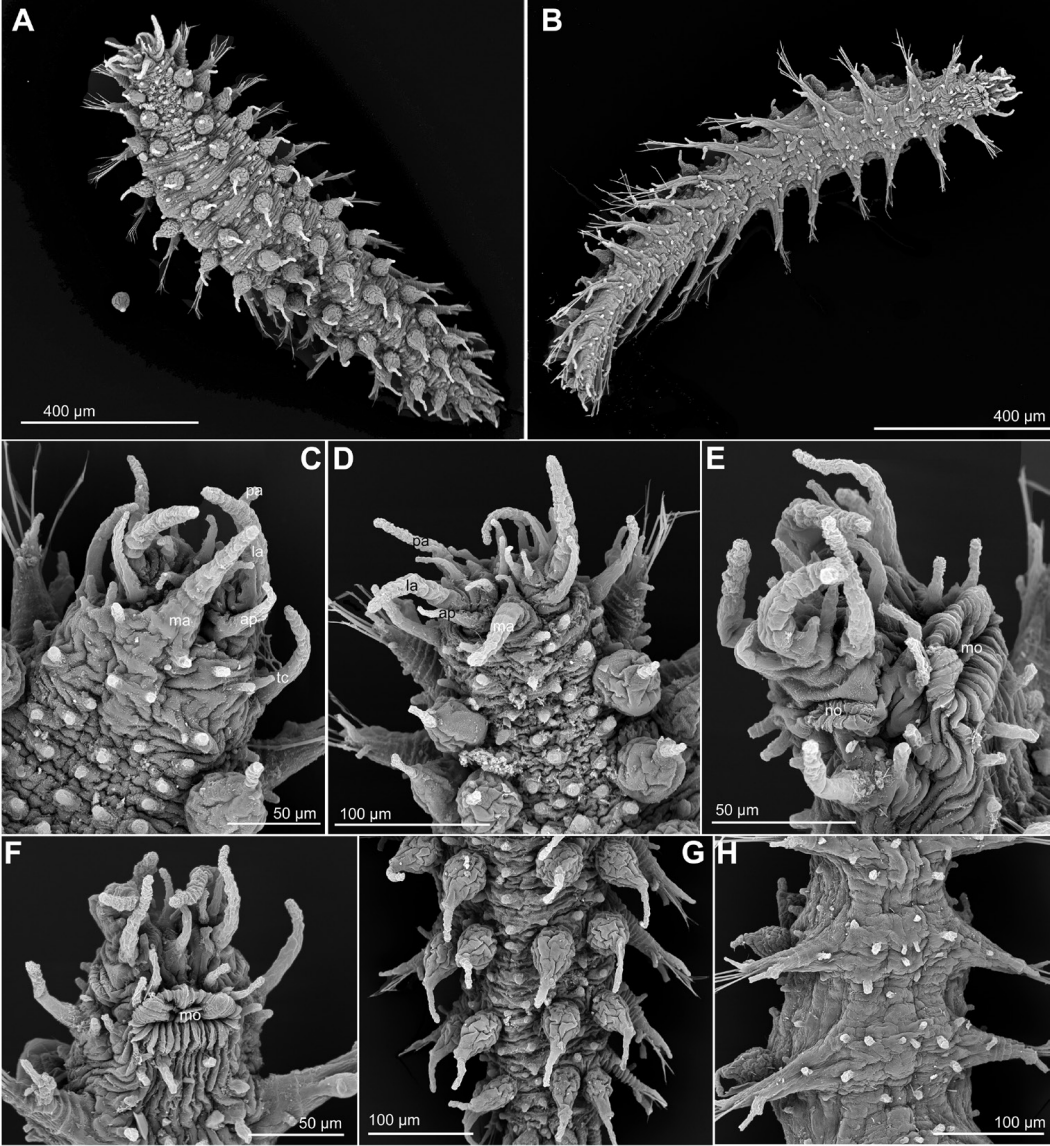
*Sphaerephesia amphorata* sp. n., paratypes, USNM 1002041, SEM. **A** Whole specimen, dorsal view **B** Same, ventral view **C** Anterior end, showing head appendages, dorsal view **D** Same, with head papillae visible **E** Same, lateroventral view; mouth and nuchal organ pits, visible **F** Anterior end, ventral view **G** Posterior chaetigers, dorsal view, showing the characteristic dorsal macrotubercles with long terminal papillae **H** Mid-body chaetigers, ventral view. Abbreviations: lateral antenna; ma, median antenna; mo, mouth; no, nuchal organ; pa, palp; tc, tentacular cirrus.

*Measurements and general morphology*. Gravid female, 1.5 mm long, 0.2 mm wide, with17 chaetigers. Body elongated, tapering at both ends, slightly flattened dorso-ventrally (wider than high). Dorsum convex and ventrum flattened (Fig. 5A, B). Tegument with transverse wrinkles and segmentation not obvious (Fig. 5A). Preserved specimen lacking pigmentation.

*Head*. Anterior end bluntly rounded (Fig. 5A, C–F). Prostomium with seven longer appendages including a pair of palps, in ventral-most position near the mouth, a pair of lateral antennae and a median antenna; and a pair of antenniform papillae behind lateral antennae, all digitiform, slightly wrinkled and similar in size except for the antenniform papillae, slightly shorter than lateral antennae (Fig. 5C–F). Approximately 10 digitiform papillae confined by prostomial appendages and mouth, in frontal view (Fig. 5D, F). A pair of tentacular cirri, similar in shape and size to lateral antennae and palps, and several scattered papillae similar to prostomial in head surroundings. Nuchal organ pits located between lateral antennae and tentacular cirri (Fig. 5E).

**Figure 6.**
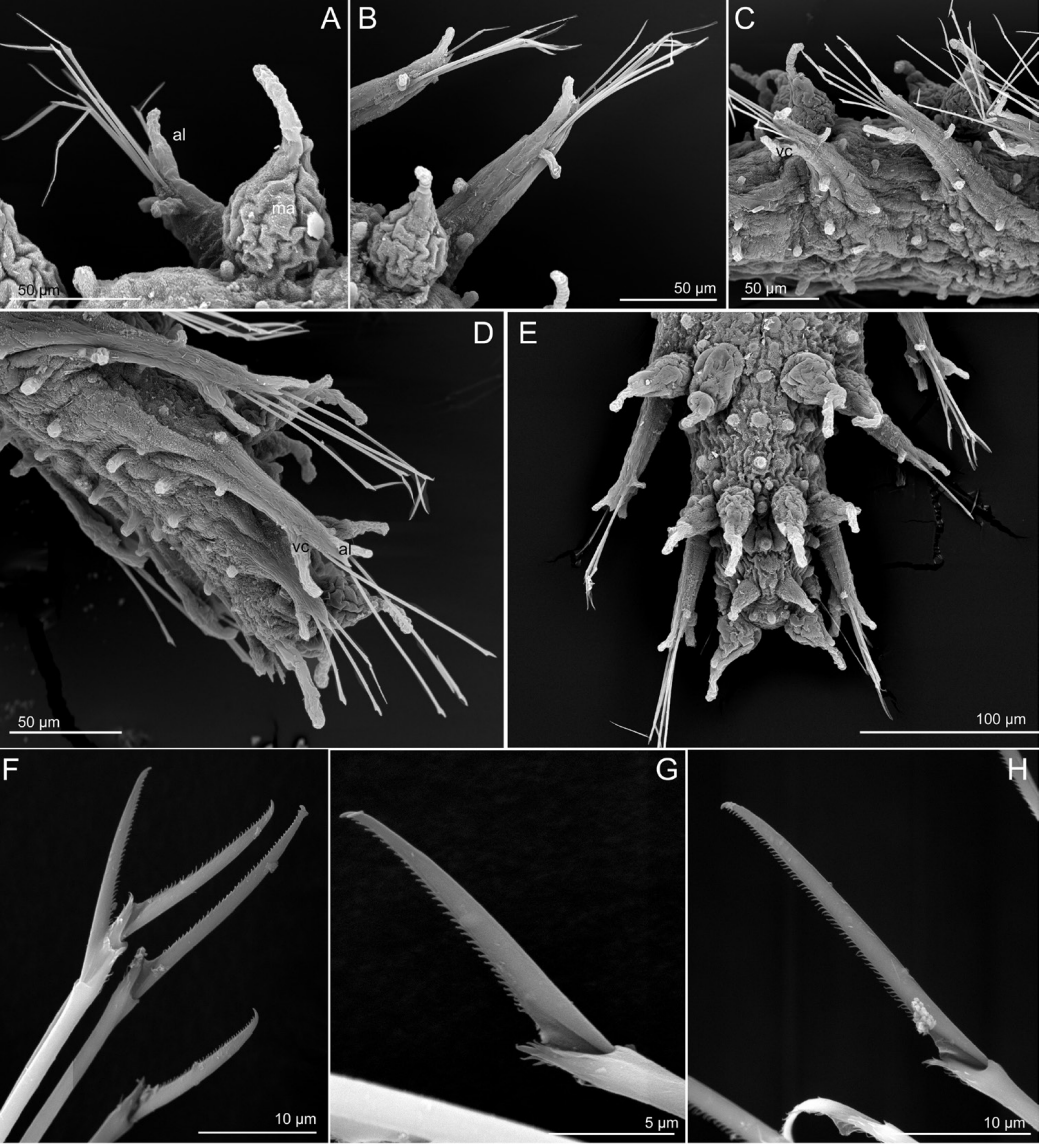
*Sphaerephesia amphorata* sp. n., paratypes, USNM 1002041, SEM. **A** Anterior parapodium, dorsal view **B** Mid-body parapodium, dorsal view **C** Mid-body chaetigers, lateroventral view **D** Posterior end, side view **E** Posterior end, dorsal view **F** Chaetae anterior parapodium **G, H** Detail of chaetae, mid-body chaetiger. Abbreviations: al, acicular lobe; mc, macrotubercle; vc, ventral cirrus.

*Tubercles*. First and last chaetigers with two macrotubercles, sessile, bottle-shaped and provided with a long terminal papilla (Figs 5A, C, D, 6E). Rest of chaetigers with four macrotubercles each, arranged in four longitudinal rows along dorsum (Figs 5C, 6A, B). Distance between mid rows and lateral rows similar (Fig. 5A–B). Shape and size of all macrotubercles similar, slightly decreasing in size in last chaetigers (Fig. 5A). Spherical or ellipsoid papillae present over dorsum, arranged in 3–4 transversal rows per segment (Fig. 5A, D, G), around 10 papillae present between mid-macrotubercles and five between these and lateral ones in mid-body segments (Fig. 5A, G). Microtubercles absent. Ventral surface with small ellipsoid papillae, arranged in 3–4 irregular transversal rows (Fig. 5H), with a total of around 12 papillae per segment in mid-body; numbers decreasing towards posterior end (Fig. 6D). Body epithelium with ellipsoid granules (e.g. Fig. 6A).

*Parapodia*. Parapodia elongated, sub-conical, increasing in size towards chaetiger 4 and around 2–3 times longer than wide (Figs 5B, H, 6A–D). Acicular lobe projecting distally anterior to chaetae (Fig. 6A–D). Ventral cirri sub-conical, pear-shaped, as long as acicular lobe in posterior chaetigers, and shorter in anterior (Figs 5B, H, 6A–E). Mid-body parapodia with about four small spherical papillae, all similar in size: one distal, on dorsal surface, one anterio-dorsal and one anterio-ventral and another ventral, all in proximal half of parapodia (Figs 5H, 6A–E).

*Chaetae*. Compound chaetae present in all chaetigers, arranged in a curved transverse row (C-shaped) behind acicular lobe and numbering 4–7 per fascicle (Fig. 6A–D). Shaft with slightly widened distal end with delicate, almost inconspicuous spinulation (Fig. 6G, H). Blades decreasing in size from mid-fascicle to dorsal and ventral ends (7–11 times longer than maximum width), with fine and short spinulation along cutting edge and a slightly curved tip (Fig. 6G, H).

*Pygidium*. Pygidium terminal, with mid-ventral digitiform anal cirrus and a pair of dorsal anal cirri, similar in shape to macrotubercles (Fig. 6E).

*Internal features*. Eyes not observed in any specimen. Muscular pharynx runs along three anterior chaetigers.

*Reproductive features*. Some paratypes and additional specimens examined are gravid females carrying large discoid eggs, 200 µm in diameter that occupy most of the body coelom, from the anterior to the posterior segments; other specimens seem to be filled with sperm. However, ‘copulatory organs’ were not observed in either females or males.

#### Variation.

Paratypes varying in size from 0.8 to 1.5 mm and seven to 17 chaetigers. Most features are conserved in this species and all specimens examined regardless the size bear the dorsal macrotubercles with the unusual elongated terminal papillae as long as the macrotubercle. Length of blades vary within fascicles and also along the chaetigers, generally between 7–11 times longer than maximum width.

#### Remarks.

The most conspicuous and distinct morphological attribute of *Sphaerephesia amphorata* sp. n. is the presence of dorsal macrotubercles with elongated papillae providing them the characteristic amphora shape, while other described species in the genus have a rounded terminal papilla. Six of the nine nominal *Sphaerephesia* species share with *Sphaerephesia amphorata* sp. n. the presence of four rows of macrotubercles with a terminal rounded papilla, several additional papillae on dorsal surface, and falcigers with long blades. These are *Sphaerephesia similisetis* Fauchald, 1972, *Sphaerephesia longisetis* Fauchald, 1972, *Sphaerephesia chilensis* Fauchald, 1974, *Sphaerephesia fauchaldi* Kudenov, 1987b, *Sphaerephesia regularis* Böggemann, 2009 and *Sphaerephesia hutchingsae* Capa & Bakken, 2015. None of the species mentioned share with the new species the number of parapodial papillae; *Sphaerephesia fauchaldi*, *Sphaerephesia hutchingsae*, *Sphaerephesia longisetis* and *Sphaerephesia similisetis* bear over six parapodial papillae while *Sphaerephesia regularis* and *Sphaerephesia chilensis* are provided with one or two parapodial papillae (Fauchald 1972, 1974, Kudenov 1987b, Capa and Bakken 2015). Other differences between *Sphaerephesia longisetis*, *Sphaerephesia fauchaldi* and *Sphaerephesia amphorata* sp. n. are the presence of microtubercles in the former two species, absent in the latter.

#### Distribution.

Only known from type locality, East of Cape Lookout, North Carolina, United States, North Atlantic, ranging from 580 to 2003 m.

#### Etymology.

Amphora, is a Greek word that refers to the pottery vases used since the Bronze Age by the Greco-Romans to transport liquids. The shape of these containers resembles the characteristic shape of the dorsal macrotubercles of this species.

### Sphaerodoridium

Taxon classificationAnimaliaPhyllodocidaSphaerodoridae

Lützen, 1961

Sphaerodoridium Lützen, 1961: 409–410 (in part), Fauchald 1974: 270, Capa et al. 2014.

#### Type species.

*Sphaerodorum claparedii* Greeff, 1866

#### Diagnosis.

Body short and ellipsoid. Six or more longitudinal rows of macrotubercles on dorsum, in one transversal row per segment. Macrotubercles stalked and smooth, without terminal papilla. Smaller, stalked tubercles on ventrum. Microtubercles absent. Papillae over body surface and parapodia. Prostomial and peristomial appendages digitiform. Parapodia with only compound chaetae; hooks absent.

#### Remarks.

Capa et al. 2016 recovered *Clavodorum* nested within *Sphaerodoridium*, indicating the circumscription of these two genera according to the relative length of the head appendages should be revised.

### Sphaerodoridium minutum

Taxon classificationAnimaliaPhyllodocidaSphaerodoridae

(Webster & Benedict, 1887)

Fig. 7

Ephesia minuta Webster & Benedict, 1887: 728–729, pl. IV, figs 64–66.
Sphaerodoropsis minuta .– Imajima 1969: 153–154, fig. 2; Imajima 2009: 77.– Hartmann-Schröder 1996: 237.– Moreira 2012: 39–41, fig. 13.
Sphaerodoum minutum .– Berkeley and Berkeley 1948: 27–28, fig. 34.
Sphaerodoridium minutum .– Lützen 1961: 415.– Capa et al. 2016: 12.

#### Material examined.

**Lectotype**: USNM 393, Eastport, Maine, United States, North Atlantic Ocean, coll. Webster, H. E. **Paralectotypes**: USNM 1407984 (11 ind. and 4 slides), Eastport, Maine, United States, North Atlantic Ocean, coll. Webster, H. E. **Paratypes**: USNM 22873 (29 ind., 3 for SEM) Eastport, Maine, United States, North Atlantic Ocean, coll. Webster, H. E.

#### Diagnosis.

Palps and lateral antennae digitiform, median antenna shorter and digitiform. Tentacular cirri digitiform. Eyes not observed. Parapodia with three (or four) parapodial papillae; compound chaetae with blades 4–5 times as long as maximum width on mid-body chaetigers.

#### Re-description.

**Figure 7.**
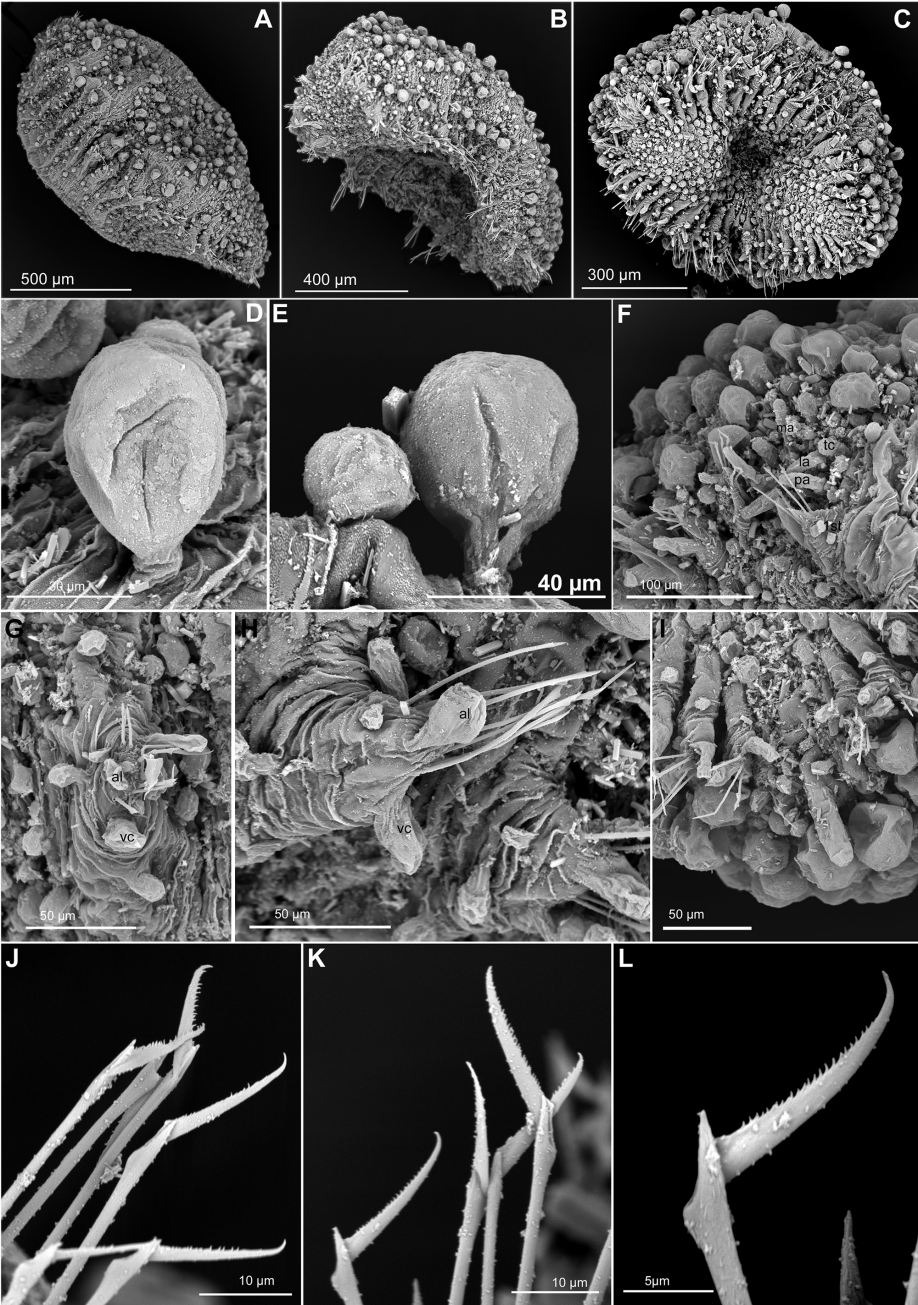
*Sphaerodoridium minutum*, paratypes USNM 22873, SEM. **A** Whole specimen, side view **B** Whole specimen, ventro-lateral view **C** Same, ventral view **D** Detail of stalked, dorsal macrotubercle **E** Dorsal macrotubercle and papilla **F** Anterior end, frontal view, showing head appendages and tubercles of anterior chaetigers **G** Mid-body parapodium, side view **H** Mid-body parapodia, anterior view **I** Posterior end and pygidium, ventral view **J** Chaetae anterior chaetigers **K** Chaetae mid-body parapodium **L** Detail of Mid-body chaeta. Abbreviations: al, acicular lobe; la, lateral antenna; ma, median antenna; mc, macrotubercle; mi, microtubercle; pa, palp; tc, tentacular cirrus; vc, ventral cirrus.

*Measurements and general morphology*. Holotype 1.1 mm long, 0.6 mm wide and with 22 chaetigers. Body ellipsoid, ovoid in cross-section, with slightly flattened ventrum and convex dorsum (Fig. 7A–C). Anterior end blunt, mid-body broad, slightly narrowing along posterior segments (Fig. 7A). Segmentation inconspicuous, tegument with transverse wrinkles (Fig. 7A–C). Preserved specimen lacking pigmentation.

*Head*. Prostomium with five short appendages, including a pair of digitiform palps in ventral-most position, a pair of digitiform lateral antennae, similar in size and shape as palps, and a digitiform median antenna, shorter than lateral antennae and palps (Fig. 7C, F). A pair of tentacular cirri shorter than lateral antennae and palps, close to lateral antennae (Fig. 7F). A few rounded small papillae scattered around head appendages (Fig. 7F).

*Tubercles*. First chaetiger with eight dorsal macrotubercles; following chaetigers each with one transversal row of dorsal macrotubercles increasing to 10–12 tubercles per segment from chaetiger 5 (Fig. 7B–C). Macrotubercles spherical to club-shaped with a short and smooth stalk (Fig. 7D–E); all macrotubercles similar in shape and size. Additional spherical and sessile papillae in different sizes over dorsum, arranged in 2–3 irregular transversal rows per chaetiger; 20–30 papillae on each mid-body chaetiger (Fig. 7A–C). Ventral surface with spherical papillae in different sizes, arranged in 2–3 transversal rows in a zig-zag pattern, with about 20 per segment in mid-body; numbers decreasing towards posterior end (Fig. 7A, C).

*Parapodia*. Parapodia sub-conical, increasing in size towards chaetiger 3 (Fig. 7C), around 2 times longer than wide (Fig. 7G, H). Acicular lobe anterior to chaetae, digitiform to clavate, longer than parapodial papillae and projecting distally (Fig. 7C, G, H). Ventral cirri digitiform projecting 1/2 to 2/3 as long as acicular lobe on anterior and mid-body segments, almost as long as in posterior segments (Fig. 7C, G, H). Parapodia with three spherical to clavate papillae: one on anterio-dorsal surface, one on anterio-basal position (Fig. 7F–H), and one on the posterior surface (Fig. 7F–G).

*Chaetae*. All parapodia with 4–7 compound chaetae, arranged in a curved transverse row around acicular lobe (Fig. 7F–I). Serrated, long blades, 4–5 times longer than maximum width, with a curved tip (Fig. 7J–L), similar throughout.

*Pygidium*. Pygidium terminal, with one mid-ventral digitiform anal cirrus projecting beyond parapodia, and one pair of clavate anal cirri, at base on median cirrus (Fig. 7I).

*Internal features*. Specimens are all opaque after fixation and preservation and internal features not observable.

*Reproductive features*. Copulatory organs or eggs not seen in type specimens.

#### Variation.

Most paratypes show small, digitiform anterior appendages but the anterior end is often retracted and these are not easily discernible. This species seems very homogenous regarding the number and arrangement of epithelial tubercles. The exact number of macrotubercles and papillae is, however, difficult to assess in the type material because some of these are detached or because when macrotubercles are inflated and the stalk allows some reorganization, the exact number of tubercles per segment is imprecise. Parapodial papillae in mid-body segments vary between three and four as in some specimens a second papillae can be observed at the base of the posterior parapodial surface (Fig. 7G). Sexual dimorphism not observed.

#### Remarks.

The original description is detailed and accurate for most characters, including those more difficult to observe. In most type specimens the dorsal macrotubercles are lost or they fall off when being handled and consequently the total number of tubercles vary from the 10–12 macrotubercles in each transverse row as stated in the original description. Macrotubercles are described as being attached to the body by a “short neck” (Webster and Benedict 1887), here interpreted as a short stalk. This feature justifies this species to be considered as a *Sphaerodoridium* and must have been overlooked in later studies dealing with this species, explaining why it was placed in *Sphaerodoropsis* for a long time (Berkeley and Berkeley 1948, Imajima 1969, Hartmann-Schröder 1996, Imajima 2009, Moreira 2012). Dorsal cirri were described as present (Webster and Benedict 1887), most likely referring to dorsal macrotubercles closest to parapodia.

*Sphaerodoridium minutum* is most similar to *Sphaerodoridium guerritai* Moreira & Parapar, 2015, described from the north eastern Atlantic, having as many dorsal macrotubercles, but *Sphaerodoridium minutum* lacks the characteristic papillae on the macrotubercle’s stalk that *Sphaerodoridium guerritai* possesses, and the latter species has long stalks compared to the short ones in *Sphaerodoridium minutum*. Further, there are clear differences between the two species in number and composition of parapodial papillae. *Sphaerodoridium minutum* is also similar to *Sphaerodoridium evgenovi* Gagaev, 2015, *Sphaerodoridium kolchaki* Gagaev, 2015, and *Sphaerodoridium kupetskii* Gagaev, 2015, all recently described from the Arctic Ocean, as they all share a similar arrangement and number of macrotubercles (Gagaev 2015). The two latter species, *Sphaerodoridium kolchaki* and *Sphaerodoridium kupetskii*, have long macrotubercle stalks, hence they are different from *Sphaerodoridium minutum*. The seemingly short macrotubercle stalks in *Sphaerodoridium evgenovi* are rather similar to those in *Sphaerodoridium minutum*. However, *Sphaerodoridium evgenovi* has chaetae with somewhat shorter blades, lack parapodial papillae on the posterior surface, and also has parapodia much longer than wide compared to *Sphaerodoridium minutum* (Gagaev 2015). The three species described by Gagaev (2015) and the one described by Moreira and Parapar (2015) are all similar to each other and show resemblance to *Sphaerodoridium minutum*. These two papers were published the same year and the authors were most likely not aware of each other’s work. These species should be compared in detail in order to assess the species delimitation (Gagaev 2015).

*Sphaerodoridium minutum* has similar number of stalked macrotubercles to *Clavodorum polypapillata* (Hartmann-Schröder & Rosenfeldt, 1988) described from Antarctica, and *Clavodorum andamanense* Bakken, 2002 described from Thailand. In *Clavodorum polypapillata* the original description overlooked stalked macrotubercles (Moreira and Parapar 2011), and has been re-described to have a larger number of macrotubercles (12–17 per transverse row, compared to 10–12 present in *Sphaerodoridium minutum*) and a large number of ventral papillae (>40 per segment compared to ca. 20 in *Sphaerodoridium minutum*). *Clavodorum polypapillata* further has longer stalks than in *Sphaerodoridium minutum* (Moreira and Parapar 2011). *Clavodorum andamanense* lacks papillae on the dorsum, unlike *Sphaerodoridium minutum*.

*Sphaerodoridium minutum* has been reported with a wide geographic distribution (Berkeley and Berkeley 1948, Lützen 1961, Imajima 1969, 2009, Hartmann-Schröder 1996). In order to clarify the geographic distribution of this species careful examination of specimens reported under this name from other geographic areas is needed.

The validity and clear delimitation of *Sphaerodoridium* and *Clavodorum* Hartman & Fauchald, 1971 has been regarded as doubtful. Several authors pointed out the insubstantial generic differences between the two genera (Bakken 2002, Moreira and Parapar 2011, 2015) and only recently reciprocal monophyly was not assessed in the first phylogenic hypothesis of the family (Capa et al. in 2016). There are currently 10 species of *Sphaerodoridium* and another 10 of *Clavodorum* described from world-wide areas and from shelf to abyssal depths (Capa et al. 2014, Moreira and Parapar 2015, Gagaev 2015). As the delimitation of the two genera is not clear, and species representing the two genera are phylogenetically nested in a group (Capa et al. 2016), it will be necessary to assess all described species in both genera when comparing similarities.

#### Distribution.

This species is known from the New England region of the US. It has been reported from the North Atlantic (e.g. Hartmann-Schröder 1996), North Pacific (e.g. Berkeley and Berkeley 1948) and Japan (Imajima 1969, 2009). The emergence of descriptions of new species similar to *Sphaerodoridium minutum* suggests a review of the true identity of reported specimens is needed.

### Sphaerodoropsis

Taxon classificationAnimaliaPhyllodocidaSphaerodoridae

Hartman & Fauchald, 1971

Sphaerodoropsis Hartman & Fauchald, 1971: 69; Fauchald 1974: 69.

#### Type species.

*Sphaerodorum sphaerulifer* Moore, 1909.

#### Diagnosis.

Body generally short and ovoid, some forms slender. Four or more longitudinal rows of macrotubercles, in one or several transverse rows per segment. Macrotubercles sessile and smooth, without terminal papillae. Microtubercles absent. Papillae over body surface and parapodia. Prostomial and peristomial appendages short, spherical or digitiform. Parapodia with compound chaetae; hooks absent.

### Sphaerodoropsis corrugata

Taxon classificationAnimaliaPhyllodocidaSphaerodoridae

Hartman & Fauchald, 1971

Figs 8 , 9

Sphaerodoropsis Hartman & Fauchald, 1971: 69–71, pl 34, figs a, b.
Sphaerodoridium sp. A. – Hartman 1965: 94, Pl. 14, fig. f.

#### Material examined.

**Holotype**: LACM-AHF POLY 950, west of Atlantis Canyon, New England continental slope, North Atlantic, 39°56.5'N, 70°39.9'W, 400 m, 28 Aug 1962. **Additional material.** USNM 1002203 (1 ind.), off Massachusetts, United States, North Atlantic Ocean, 40°01.284'N, 70°55.032'W, 255 m, 8 Dec 1984; USNM 1002207 (6 ind., 3 for SEM) Lydonia Canyon, Georges Bank, United States, 40°21.114'N, 67°32.232'W, 590 m, 28 Apr 1985; USNM 1002209 (4 ind.) ff Massachusetts, United States, 40°01.248'N, 70°55.086'W, 250 m, 4 May 1985; USNM 1002193 (2 ind., 1 for SEM), Off Cape Hatteras, North Carolina, United States, 35°26.268'N, 74°41.436'W, 2003 m, 24 May 1985; USNM 1002212 (1 ind.) off Massachusetts, United States, 39°50.382'N, 70°01.65'W, 1239 m, 27 Nov 1985; USNM 1001989 (2 juvenile ind.) off New Jersey, United States, 38°40.068'N, -072°56.418'W, 1519 m, 13 Nov 1985; USNM 1001830 (1 ind.), Baltimore Canyon, United States, 37°53.286'N, 73°45.264'W, 1619 m, 7 Aug 1984; USNM 1002032 (1 ind.), off Massachusetts, 39°48.36'N, 70°54.93'W, 1249 m, coll. Battelle-New England Marine Research Lab For BLM/ MMS, 30 Nov 1985; USNM 1002033 (1 ind.), Georges Bank, 41°01.35'N, 66°20.23'W, 1345 m, coll. Battelle-New England Marine Lab For BLM/ MMS, 25 Jul 1986; USNM 1002035 (1 ind.), Georges Bank, 41°01.54'N, 66°20.11'W, 1345 m, coll. Battelle-New England Marine Lab For BLM/ MMS, 25 Jul 1986.

#### Comparative material.

*Sphaerephesia fauchaldi* Kudenov, 1987b, holotype NMNH 102785; *Sphaerephesia longisetis* Fauchald, 1972, holotype AHF POLY 0964; *Sphaerephesia regularis* Böggemann, 2009, holotype ZMH P25498, paratypes ZMH P25497 (4 ind.), ZMH P25499 (4 ind.); *Sphaerephesia similisetis* Fauchald, 1972, paratype AHF POLY 0967 (2 ind.). *Sphaerephesia hutchingsae* Capa & Bakken, 2015, holotype East of Malabar, Sydney, New South Wales, Australia, AM W.42748, 33°58.71667'S, 70°39.9'E, 82 m, 22 Aug 1995; paratypes from nearby collecting sites (see Capa and Bakken 2015).

#### Diagnosis.

Body ellipsoid, with four longitudinal rows of sessile, rounded to pear-shaped macrotubercles without terminal papillae, per segment. Distance between dorsal-most macrotubercles exceeds distance between those and lateral ones. Parapodia with ventral cirri as long as acicular lobe or slightly shorter and 4–6 rounded, small papillae, sometimes a dorsal one slightly larger. Parapodia with 10–16 compound chaetae, with thin shafts and blades 12–16 times as long as wide.

#### Re-description.

*Measurements and general morphology*. Gravid female, 2 mm long, 0.5 mm wide, with 17 chaetigers. Body ellipsoid, slightly flattened dorso-ventrally (wider than high). Tegument with transverse wrinkles and segmentation only barely discernible. Preserved specimen lacking pigmentation.

**Figure 8.**
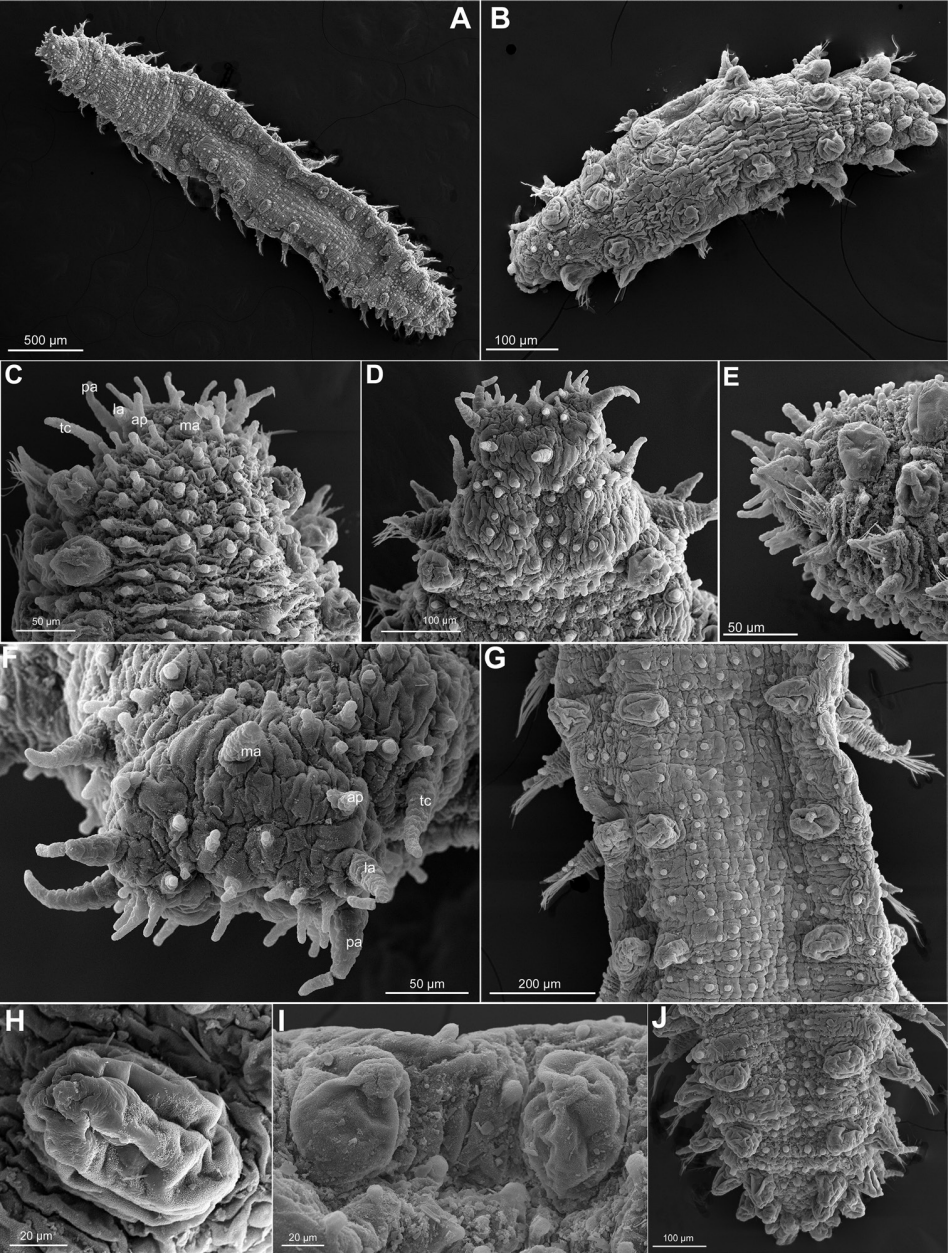
*Sphaerodoropsis corrugata*, USNM 1002193 and 1002207. **A** Whole specimen, dorsal view **B** Juvenile, dorsal view **C, D** Anterior end, dorsal view **E** Same, side view **F** Head, frontal view **G** Mid-body segments, dorsal view **H, I** Mid-body dorsal macrotubercles **J** Posterior segments and pygidium, dorsal view. Abbreviations: la, lateral antenna; ma, median antenna; pa, palp; tc, tentacular cirrus.

*Head*. Anterior end bluntly rounded (Fig. 8A–F). Prostomium with seven longer appendages, including a pair of palps, in ventral-most position near the mouth, conical, wrinkled and about 4–5 times longer than wide at base; a pair of lateral antennae, similar in shape and size to palps; a median antenna, shorter (two thirds) than lateral antennae and with a rounded distal end; and a pair of antenniform papillae behind lateral antennae, similar in shape and size to median antenna, or smaller (Fig. 8C–F). Around 20 digitiform smaller papillae confined by prostomial appendages and mouth, in frontal view (Fig. 8F). One pair of tentacular cirri similar in shape and size to lateral antennae and palps.

*Tubercles*. First chaetiger with two macrotubercles; rest of chaetigers with four macrotubercles, each arranged in four longitudinal rows along dorsum (Fig. 8A–D, G). Distance between mid-rows larger than between these and lateral rows of macrotubercles (Fig. 8G). Size of all macrotubercles similar, with base as large as base of parapodia or smaller, or slightly increasing in size in first four chaetigers (Fig. 8A–B), also slightly reducing in size in posterior chaetigers towards pygidium. Macrotubercles spherical or pear shaped, with some pores (Fig. 8G–J). Spherical papillae present over dorsum and ventrum, with arrangement hard to determine in holotype.

**Figure 9.**
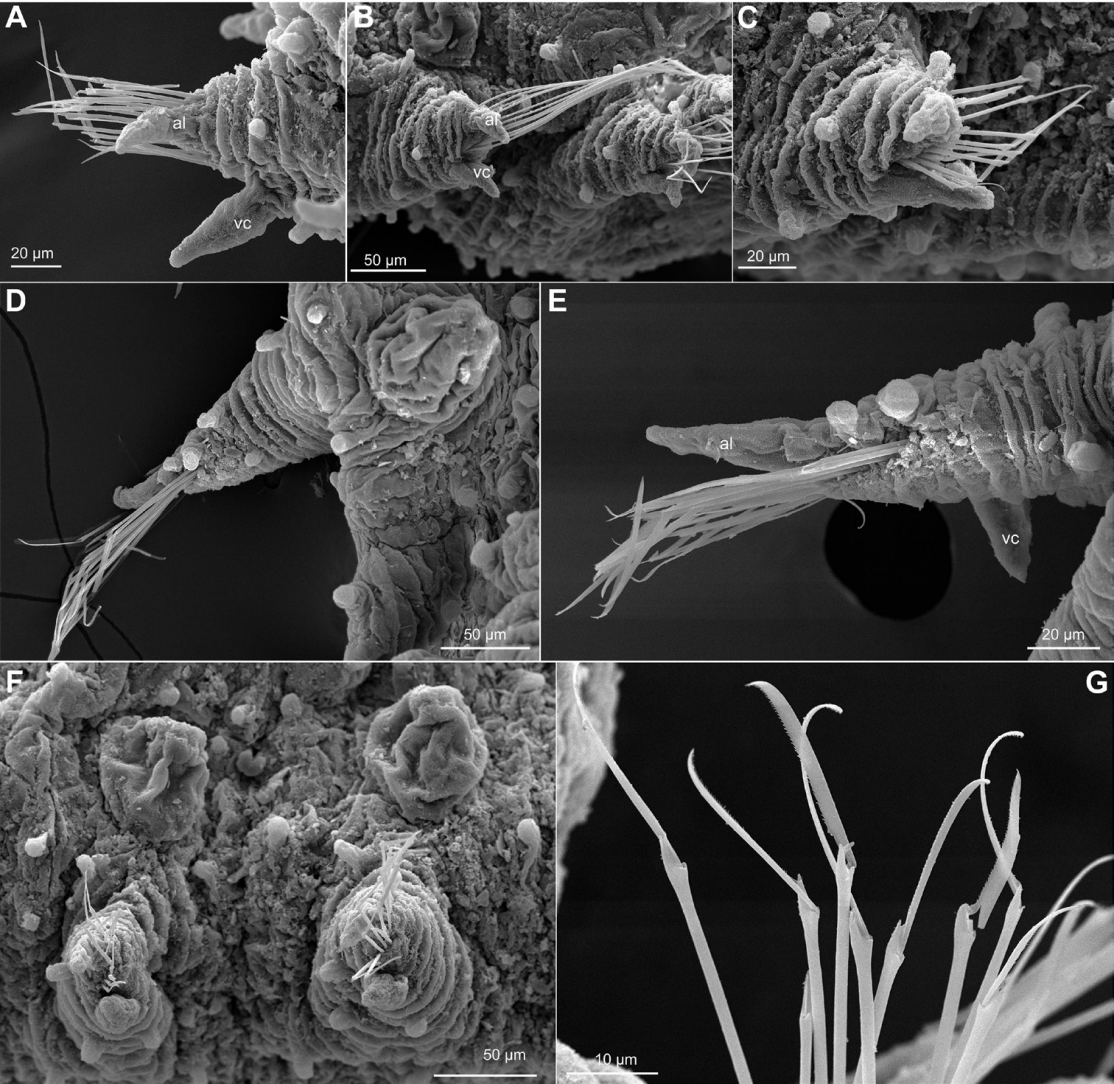
*Sphaerodoropsis corrugata*, USNM 1002193 and 1002207. **A** Parapodium first chaetiger, anterior view **B** Parapodia chaetiger 6 and 7, anterior view **C** Posterior parapodium, anterior view **D, E** Mid-body parapodia, dorsal view **F** Mid-body segments, side view **G** Mid-body chaetae. Abbreviations: al, acicular lobe; vc, ventral cirrus.

*Parapodia*. Parapodia sub-conical, 1–2 times longer than wide (shorter in anterior and posterior most chaetigers), wrinkled (Fig. 9A–E). Acicular lobe projecting distally anterior to chaetae (Fig. 9A–E). Ventral cirri sub-conical, similar in length to acicular lobe but not projecting over the tip of acicular lobe (Fig. 9A). Mid-body parapodia with 4–5 spherical papillae: 1–2 on dorsal surface, 1 on anterior surface, 2 on ventral surface and 0–1 on posterior surface (Fig. 9A–F); all similar in size or a dorsal slightly larger.

*Chaetae*. Compound chaetae present in all chaetigers, arranged in a curved transverse row around acicular lobe and numbering 8–12 per fascicle in mid-body chaetigers (Fig. 9A–E). Blades similar in length along fascicles (12–16 times longer than maximum width), only slightly longer than those from mid-fascicle, with fine and short spinulation along superior edge, slightly curved (Fig. 9G).

*Pygidium*. Pygidium terminal, with mid-ventral digitiform anal cirrus and a pair of dorsal anal cirri, similar in shape to macrotubercles but slightly smaller.

*Internal features*. Eyes not observed in any specimen; holotype with a pair of reddish anterior spots that may not be eyes, but the nuchal organs. Muscular pharynx runs along chaetigers 3–6.

*Reproductive features*. Holotype and a few of the additional specimens examined are gravid females carrying large discoid eggs, 200 µm in diameter that occupy most of the body coelom, from the anterior to the posterior segments. “Copulatory organs” not observed.

#### Variation.

Specimens measured from 0.6 mm to 3.5 mm. Specimens assigned to this species show some variation in the number and arrangement of the epithelial papillae, probably because there are not easily ascertained in the holotype and were not described in detail in the original description. Larger specimens with approximately 30 papillae present between mid-macrotubercles and 10 between these and the lateral ones in mid-segments (Fig. 8G). Smaller specimens with less epithelial papillae (Fig. 8B). About five papillae between lateral macrotubercles and parapodia. Ventral surface with small papillae, arranged in about five transversal rows in mid-body. Body epithelium with ellipsoid granules (Fig. 8H). Chaetae numbering up to 16 per fascicle in mid-body chaetigers. Shaft with slightly widened distal end with delicate, almost inconspicuous spinulation (Fig. 9G), length of blades up to 15–16 times longer than wide.

#### Remarks.

*Sphaerodoropsis corrugata* has not been reported since its original description. Records of this species include off New York state, from 400 to 1500 m (Hartman and Fauchald 1971). The type is not in excellent conditions to examine the number and arrangement of papilla. Nevertheless, some features observed on this specimen do not match the original description or are somehow imprecise. Antenniform papillae are present, unlike indicated in original and later descriptions by Hartman and Fauchald (1971) and Fauchald (1974). The parapodial papillae are all spherical or hemispherical, there is no truncate forms (Hartman and Fauchald 1971: Fig. 34B, Fauchald 1974) and if they were observed with that shape it could have been due to a collapse of the structure that has recovered its original shape after years of preservation (see Capa and Bakken 2015 for other examples). The chaetae drawn (Hartman 1965) may be ones with shorter blades in the fascicle, and some of the additional chaetae observed in the type and additional material are provided with longer and slender blades. As originally described, the acicular lobe is well developed and there is no additional parapodial lobes, only epithelial papillae, what seems to be the common pattern from all members in the family (Capa et al. 2014), and not characteristic of this particular species. The different terminology for papillae used by different authors creates confusion.

*Sphaerodoropsis elegans* Hartman & Fauchald, 1971 originally described from Brazil but also reported from New England, resembles *Sphaerodoropsis corrugata*. It also belongs to the *Sphaerodoropsis* Group 1 *sensu* Borowski (1994) and is provided with long blade chaetae. Review of the types of this species reveal that the macrotubercles are pear-shaped, almost possessing a terminal papilla, indicating they could formally be considered as *Sphaerephesia*. Other *Sphaerephesia* species with ellipsoid body, four rows of macrotubercles with a terminal rounded papilla, several additional papillae on dorsal surface, and falcigers with long blades are *Sphaerephesia similisetis* Fauchald, 1972, *Sphaerephesia longisetis* Fauchald, 1972, *Sphaerephesia chilensis* Fauchald, 1974, *Sphaerephesia fauchaldi* Kudenov, 1987b, and *Sphaerephesia hutchingsae* Capa & Bakken, 2015. *Sphaerodopsis corrugata* differs from all of these in the number of parapodial papillae since the other have one or two (*Sphaerodopsis chilensis*) or more than seven (Fauchald 1972, 1974, Kudenov 1987b, Capa and Bakken 2015) while *Sphaerodopsis corrugata* has four or five in mid-body chaetigers.

#### Distribution.

New England, United States, 250–2000 m.

### Key to sphaerodorids reported from shelf and continental slope environments in the northwest Atlantic between the Artic and the equator

The species reported from the northwestern Atlantic (considered herein as the continental shelf and slope areas off Atlantic Canada and New England) are marked with *.

**Table:** 

1	Long-bodied individual, with two longitudinal rows of spherical macrotubercles (large tubercles) on dorsum with a terminal papilla, and two additional rows of microtubercles (small tubercles with a collar and a terminal papillae) running along the body within the macrotubercles rows	**2**
–	Body ellipsoid with blunt anterior and posterior ends, dorsal tubercles in more than two longitudinal rows, dorsal microtubercles absent	**5**
2	All chaetae simple, robust, with hooked distal ends and rounded edges	*Sphaerodorum* “*flavum*” Ørsted, 1843*
–	At least some chaetae compound or semi compound	**3**
3	All parapodia, except in first or second anterior segment, bearing compound and simple chaetae, the latter with angled edges	***Ephesiopsis guayanae* Hartman & Fauchald, 1971** *
–	All chaetae, except in first or second anterior segment, compound or semi-compound, often with lost blades	**4**
4	Parapodia with 5‒6 papillae each, all arranged at distal end, no erect papilla on dorsal surface of parapodia	*Ephesiella macrocirris* Hartman & Fauchald, 1971*
–	Parapodia with two papillae each, not at distal end, one erect papilla on dorsal surface and one on anterior surface of each parapodium	*Ephesiella mixta* Hartman & Fauchald, 1971*
–	Parapodia with about 11 papillae each, two on superior margin of parapodia, in addition to others (about nine) distributed randomly over the parapodia	***Ephesiella bipapillatum* Kudenov, 1987**
5	Dorsal macrotubercles, stalked, arranged in six or more longitudinal rows	**6**
–	Dorsal macrotubercles sessile, arranged in four or more longitudinal rows	**9**
6	Lateral antennae without spurs at their bases, ventrum with 10 or more longitudinal rows of papillae in mid-body segments	**7**
–	Lateral antenna with spurs at their bases, ventrum with up to six longitudinal rows of papillae in mid-body segments	**8**
7	Antennae over three times longer than wide, middle antennae often longer than lateral antennae. Ventrum with 10 longitudinal rows of papillae in middle segments, parapodia with 3–6 papillae	***Clavodorum mexicanum* Kudenov, 1987**
–	Antennae short, up to three times longer than wide, middle antennae often shorter than lateral. Ventrum with up to 15 longitudinal rows of papillae in middle segments, parapodia with 2–3 papillae	***Sphaerodoridium minutum* (Webster & Benedict, 1887)** *
8)	Ventrum with papillae arranged in two longitudinal rows, parapodia with a single papilla on the ventral surface	*Clavodorum atlanticum* Hartman & Fauchald, 1971*
–	Ventrum with papillae arranged in six longitudinal rows, parapodia with four papillae	***Sphaerodoridium lutzeni* Kudenov, 1987**
9	Dorsal macrotubercles arranged in four longitudinal rows, and one transverse line per segment; chaetae compound	**10**
–	Dorsal tubercles, considered papillae because of the smaller size compared to macrotubercles of other species, arranged in over 10 longitudinal rows, and approximately two transverse rows per segment; chaetae simple, widened subdistally	***Euritmia carolensis* sp. n.** *
10	Dorsal macrotubercles smooth, mainly spherical but some, especially in posterior segments can be pear-shaped	**11**
–	Dorsal macrotubercles with a terminal papilla	**12**
11	Parapodia with 4–6 rounded, small papillae, sometimes a dorsal one slightly larger, 10–16 compound chaetae with blades 12–16 times as long as wide	*Sphaerodoropsis corrugata* Hartman & Fauchald, 1971*
–	Parapodia with about 20 papillae and up to 10 chaetae about eight times as long as wide	***Sphaerodoropsis vittori* Kudenov, 1987**
12	Parapodia with a single papilla near de base of the superior edge; chaetae are about eight times as long as wide	*Sphaerodoropsis elegans* Hartman & Fauchald, 1971*
–	Parapodia with four papillae; 7–9 chaetae with blades 10–12 times as long as wide	*Sphaerodoropsis longipalpa* Hartman & Fauchald, 1971*

## Supplementary Material

XML Treatment for Ephesiopsis

XML Treatment for Ephesiopsis guayanae

XML Treatment for Euritmia

XML Treatment for Euritmia carolensis

XML Treatment for Sphaerephesia

XML Treatment for Sphaerephesia amphorata

XML Treatment for Sphaerodoridium

XML Treatment for Sphaerodoridium minutum

XML Treatment for Sphaerodoropsis

XML Treatment for Sphaerodoropsis corrugata
